# Diagnosis disclosure to adolescents living with HIV in rural Kenya improves antiretroviral therapy adherence and immunologic outcomes: A retrospective cohort study

**DOI:** 10.1371/journal.pone.0183180

**Published:** 2017-10-09

**Authors:** Gregg Joseph Montalto, Fredrick K. Sawe, Argwings Miruka, Jonah Maswai, Ignatius Kiptoo, Appolonia Aoko, Chrispine Oreyo, Eunice Obiero, Sheila Korir, Stephen K. Bii, Katherine X. Song, Anjali N. Kunz

**Affiliations:** 1 Division of Adolescent Medicine, Department of Pediatrics, Naval Medical Center San Diego, San Diego, California, United States of America; 2 Kenya Medical Research Institute, Walter Reed Project, Kericho, Kenya; 3 Ministry of Medical Services, Kericho District Hospital, Kericho, Kenya; 4 United States Military HIV Research Program, Rockville, Maryland, United States of America; 5 Department of Pediatrics, Madigan Army Medical Center, Joint Base Lewis-McChord, Washington, United States of America; International AIDS Vaccine Initiative, UNITED STATES

## Abstract

**Background & aims:**

Emphasis on adolescent HIV has increased worldwide as antiretroviral treatment has greatly extended life expectancies of HIV-positive children. Few evidence-based guidelines exist on the optimal time to disclose to an adolescent living with HIV (ALHIV); little is known about the medical effects of disclosure. This study looked to determine whether disclosure is associated with improved medical outcomes in ALHIV. Prior work has tended to be qualitative, cross-sectional, and with an emphasis on psychosocial outcomes. This paper addresses the adolescent cohort retrospectively (longitudinally), building upon what is already known about disclosure.

**Methods:**

Retrospective, longitudinal clinical record reviews of ALHIV seen at Kericho District Hospital between April 2004 and November 2012 were performed. Patient demographics and clinical outcomes were systematically extracted. The student’s t-test was used to calculate changes in mean CD4 count, antiretroviral therapy (ART), and cotrimoxazole adherence pre- vs. post-disclosure. Linear regression modelling assessed for trends in those clinical outcomes associated with age of disclosure.

**Results:**

Ninety-six ALHIV (54 female, 42 male) were included; most (73%) entered care through the outpatient department. Nearly half were cared for by parents, and 20% experienced a change in their primary caregiver. The mean time in the study was 2.47 years; mean number of visits 10.97 per patient over the mean time in the study. Mean disclosure age was 12.34 years. An increase in mean ART adherence percentage was found with disclosure (0.802 vs. 0.917; p = 0.0015). Younger disclosure age was associated with significantly higher mean CD4 counts over the course of the study (p = 0.001), and a nonsignificant trend toward a higher mean ART adherence percentage (p = 0.055).

**Conclusion:**

ART adherence and improved immunologic status are both associated with disclosure of HIV infection to adolescent patients. Disclosure of an HIV diagnosis to an adolescent is an important means to improve HIV care.

## Introduction

Adolescents and young adults account for 40% of the world’s new HIV infections, and remain a socially and economically vulnerable population[1]. In 2012 in Kenya, youth (defined by WHO as between the ages of 15–24 years of age)–who comprise 20.6% of the Kenyan population[2]–had an overall HIV infection prevalence of 2.1%, with a nearly 3-to-1 female-to-male prevalence ratio[3]. The majority of the population in Kenya becomes sexually active between the ages of 15 and 19 years. One in five youth aged 15–24 years reported a sexual debut before the age of 15, with fewer than two-thirds of those youth reporting using a condom the first time they had intercourse. Further, as sexual relationships build, 89% of young women abandon condom use[4]. Targeting adolescents and young adults for strategies involving HIV prevention and treatment is essential for any national program’s success[5,6].

Despite these risk factors and for HIV transmission and acquisition, challenges to effective adolescent antiretroviral therapy (ART) are encountered in low- and middle-income countries. Such challenges include psychosocial issues such as peer pressure, the stigma of HIV infection, and school-related concerns, as well as poor medication adherence[6–9]. Adherence to ART has been shown to be lower in adolescents than in adults in southern Africa[7]. Similar treatment and adherence concerns are found among adolescent patients in all socioeconomic classes worldwide and with all types of chronic illness[10].

Opportunities exist to affect adolescents’ and emerging young adults’ prognosis, through their own understanding and ownership of a disease process. Depending on an individual adolescent’s resiliency, support network and resources, knowledge of his or her own HIV infection can either be remarkably valuable or potentially devastating. Specific knowledge of one’s own HIV status–*disclosure*–has been looked at somewhat extensively vis-à-vis the effect it has on access to and retention in care, on how and when the disclosure process should be performed, and on the psychosocial benefits of disclosure to the pediatric or adolescent patient[7,11–19]. How and when to disclose an HIV diagnosis to a child or adolescent is discussed worldwide[11–14,20–24]; however, few evidence-based guidelines exist on the optimal time and circumstances by which to disclose an HIV diagnosis to an infected adolescent[24], and little is known about the medical effects of disclosure.

To date, most studies have been primarily qualitative psychosocial assessments, and few have focused on medical outcomes. Quantitative studies and outcomes data are far less prevalent; one study done in Romania showed that a patient’s knowledge of his or her HIV infection was related to delayed disease progression[23], while an adherence study in Zambia showed a positive correlation between adherence to ART and knowledge of one’s own HIV infection in young adolescents[9]. A recent study in West Africa showed an improved retention in care when adolescents were aware of their HIV status[25], and a cross-sectional study in South Africa showed that early and full disclosure was strongly associated with ART adherence[26]. Given the paucity of research on the medical benefits or drawbacks to HIV disclosure to adolescents, and the growing number of ALHIV, there is an urgent need to assess the medical outcomes of disclosure of HIV infection to those patients.

Developing countries and regions may still be hampered by infrastructure and resource deficits, which could serve to amplify difficulties with adolescent ART adherence. Donor nation supply channels and recent donor nation austerity measures may impact ART availability. Although non-availability of first-line antiretroviral (ARV) medications is not common in public (e.g. KDH) or established private (e.g. Unilever Central Hospital) treatment facilities in Kenya, stockouts have been reported in 34% of free-standing clinics and 43% of NGO-led facilities[27]. “Youth-friendly health services” is a relatively new concept worldwide. Lack of adolescent-specific medical care is known to decrease adolescent patient adherence[6,7]. During the retrospective study period, in the Kericho District of western Kenya, there were no adolescent-specific healthcare services associated with HIV care; however, the importance of addressing the unique needs of adolescent patients is well understood there. Discussing the appropriate time and means of disclosing an HIV diagnosis to an infected adolescent should be part of any adolescent health policy and curriculum; medical outcome data is needed to further inform this discussion.

To that end, this study aims to determine whether disclosure is associated with improved medical outcomes in ALHIV.

## Methods

### Study setting

The United States Army Medical Research Unit—Kenya/Walter Reed Project (USAMRU—K/WRP) collaborated with the Kenya Minstry of Health (MOH) and the Kenya Medical Research Institute (KEMRI) and initiated an HIV care and treatment program in the South Rift Valley Province of Kenya in 2004. This HIV care and treatment program’s objective was to support and improve existing health care systems to provide both medical and psychosocial care to HIV-infected patients, including children and adolescents. Currently the program supports eleven district-level HIV treatment hospitals in the South Rift Valley Province, including Kericho District Hospital (KDH), a public hospital which was the site of the study. KDH serves as a referral center for smaller health facilities in the region.

### Study population

The study population included all HIV-infected persons who were enrolled in the KDH clinics, who were between the ages of 9 and 19 years during the retrospective study period, and who were enrolled for at least one year during that age range. Other inclusion criteria were documented patient care in the KDH pediatric HIV clinics between 1 April 2004 and 1 November 2012, and at least one predisclosure and one postdisclosure visit. Patients who did not have a documented disclosure date were excluded.

### Data collection

This retrospective, coded clinical chart review aimed to describe the disclosure status, characteristics and outcomes of adolescents who were enrolled in the KDH HIV clinics. Individual medical records were reviewed, and study variables consolidated on a data collection form, which was de-identified by design. The KDH information technology chief, and KDH data clerks, who were not part of the study, were the only personnel with access to the protected key linking data collection forms to medical records. No study personnel had access to this link. All databases were password protected. Analyses proceeded on a coded and protected study database that was formed by extraction of data from individual outpatient medical records kept at the outpatient HIV clinic in Kericho. To ensure data was veritable, ten percent of data extractions were reviewed with the patient record for accuracy.

Variables extracted and studied included: 1) study ID; 2) clinic enrollment date; 3) sex; 4) date of birth; 5) date of death, when applicable; 6) entry point to care; 7) primary caregiver; 8) HIV diagnosis disclosure status; 9) date of disclosure, when applicable; 10) body mass index (BMI) ratio, calculated as the patient BMI relative to the 50^th^ percentile for sex and age; 11) absolute CD4 count; 12) viral load; 13) hemoglobin ratio, calculated as the patient hemoglobin relative to the 50^th^ percentile for sex and age; 14) ART adherence; and 15) presence of additional diagnoses, with description when available. Measurement of ART adherence involved caretaker or patient self-report through clinical interviews, standardized with the Morisky Medication Adherence Scale[28], and an objective assessment through pill counts and pharmacy refill records.

### Disclosure

A systematic process of full disclosure is incorporated into the management of the adolescent patients at KDH: 1) the age of the adolescent guides when and how disclosure is done; 2) baseline and updated information is gathered as to what the adolescent knows about his or her clinical management; 3) the healthcare team ascertains the relationship of the caregiver to the adolescent patient, which helps to support the adolescent if disclosure is done during the visit; 4) the healthcare team supports the caregiver if disclosing the HIV status during the clinic visit; 5) the patient is educated on ailments that are managed in the hospital; 6) HIV/AIDS and modes of transmission are discussed with the adolescent; 7) during disclosure, the adolescent is linked to support groups that are held after the scheduled clinic visit. There, he or she is involved in activities that are geared toward patient education and coping with long-term treatment.

### Data analysis

Data analysis on the coded study database was done using Stata 13 (StataCorps, College Station, Texas). Demographic data was captured and reviewed for patterns. Disclosure age was calculated to be the age of the patient at the first visit marked “disclosed.” Adherence to cotrimoxazole and ART was calculated as the percentage of visits the patient was deemed to be adherent per the standardized clinical adherence tools. A t-test was used to calculate differences between patient mean cotrimoxazole adherence percentage, mean ART adherence percentage, mean CD4 count, and mean viral load, before and after disclosure. Finally, linear regression was used to look at relationships between disclosure age and mean ART adherence percentage and mean CD4 count, while controlling for demographic variables.

### Ethical considerations

The study was conducted according to the Declaration of Helsinki, local regulatory requirements, Protection of Human Subjects (45 CFR 46), and DoD regulations on the protection of human subjects (32 CFR 219). The protocol was reviewed and approved by the institutional review boards (IRB) or independent ethics committees of the Kenya Medical Research Institute, which includes the Center for Clinical Research Scientific Committee, the Kenya Medical Research Institute (KEMRI) Scientific Steering Committee, and the KEMRI National Ethics Review Committee; and the Walter Reed Army Institute for Research (WRAIR) IRB. To ensure confidentiality, only a password-protected and de-identified electronic database was used for data analysis, with the protected patient-study ID key never available to study personnel, or anyone outside of the data clerks and KDH IT chief. As no human subjects were contacted during the retrospective study, with the strict procedures in place to maintain a de-identified database, since consent would necessarily compromise nondisclosure status to those patients unaware of their HIV infection, and since the study presented no more than minimal risk of harm to subjects, KEMRI and WRAIR justified a waiver of informed consent to the study.

## Results

Ninety-six patients met inclusion criteria and were considered in the analysis (Table 1). Among the 96 adolescents, 54 (56.25%) were female. Most patients entered care through the outpatient department (72.92%); a small minority did not have an entry point documented (16.67%). Slightly more than half were cared for by a parent (58.33%), with most of the remainder cared for by another family member. Patients ranged in age from 9.21 to 17.05 years, with a mean of 12.34. Average time in study was 2.47 years, with a range of 0.50–4.30 years.

**Table 1:** 

Demographics.		
**Sex**	Frequency	Percent
Male	42	43.75
Female	54	56.25
Total	96	100
**Entry Point**	Frequency	Percent
Outpatient Department	70	72.92
Inpatient Department	3	3.12
Voluntary Counseling and Testing	6	6.25
Not Documented	16	16.67
Unknown	1	1.04
Total	96	100
**Primary Caregiver**	Frequency	Percent
Mother	29	30.21
Father	19	19.79
Both Parents	8	8.33
Sister	8	8.33
Brother	2	2.08
Grandparent	12	12.5
Auntie	7	7.29
Uncle	7	7.29
Organization	2	2.08
Unknown	2	2.08
Total	96	100
**Change of Caregiver**	Frequency	Percent
No	77	80.21
Yes	19	19.79
Total	96	100
**Disclosure Age (years, n = 96)**		
Mean (SD)	Range	
12.34 (1.90)	9.21–17.05	
**Total Visits (n = 96)**		
Mean (SD)	Range	
10.97 (3.25)	3–14	
Ranges	n (%)	
< 5	4 (4.2%)	
5–10	32 (33.3%)	
> 10	60 (62.5%)	
**Total Time in Study (years, n = 96)**		
Mean (SD)	Range	
2.47 (0.87)	0.50–4.30	
Ranges	n (%)	
< 1	7 (7.3%)	
1–2	22 (22.9%)	
> 2	22 (22.9%)	

Table 2 shows differences in outcome variables pre- versus post-disclosure. Patient mean ART adherence percentage (n = 84) improved with disclosure, from a mean of 0.802 pre-disclosure to 0.917 post-disclosure (p = 0.0015). No differences were noted between pre- and post-disclosure mean cotrimoxazole adherence percentage (n = 96), or pre- and post-disclosure CD4 count (n = 56). Too few viral loads were obtained to adequately look for differences with disclosure.

**Table 2:** 

Paired t-tests, Pre- and Postdisclosure					
Variable	Obs	Mean	SD		95% CI
***Mean Predisclosure ARV Adherence***	84	0.80181	0.35399	**p = 0.0015**	0.72499–0.87864
***Mean Postdisclosure ARV Adherence***	84	0.91667	0.23569		0.86552–0.96782
***Mean Predisclosure CD4***	56	582.29	331.33	p = 0.2374	493.56–671.02
***Mean Postdisclosure CD4***	56	623.94	319.77		538.30–709.57
***Mean Predisclosure Septrin Adherence***	96	0.96855	0.11292	p = 0.4383	0.94567–0.99143
***Mean Postdisclosure Septrin Adherence***	96	0.95520	0.15922		0.92294–0.98746

**Fig 1:**
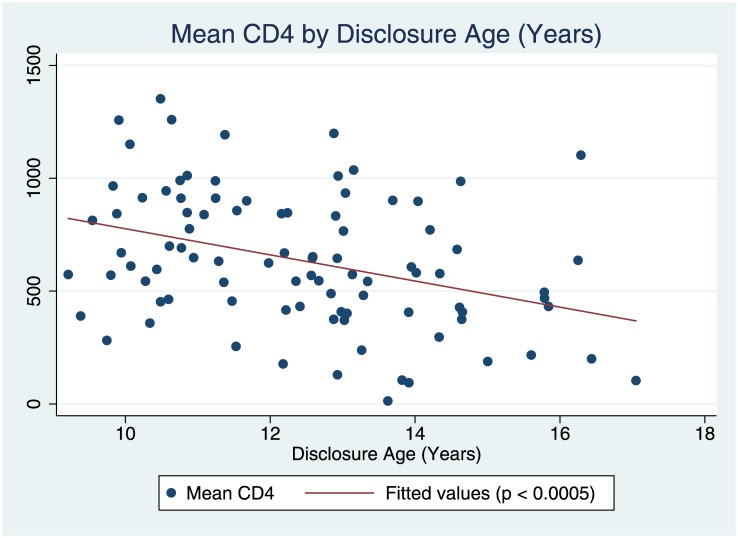
Mean CD4 by disclosure age (years).

**Fig 2:**
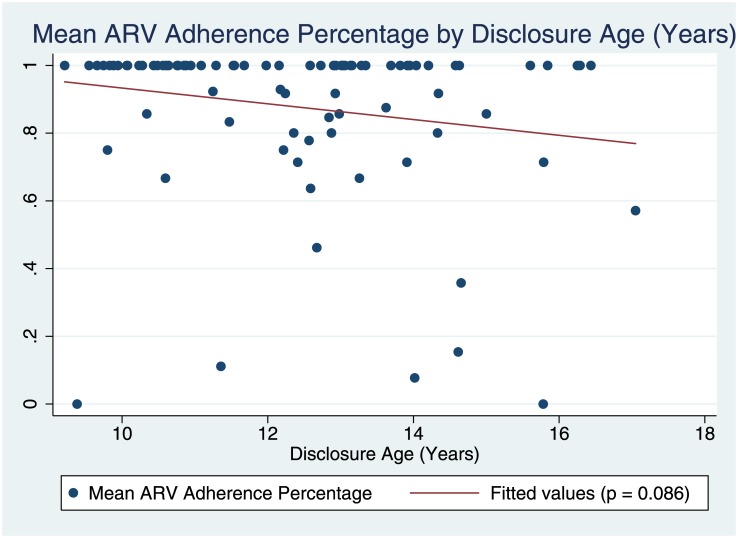
Mean ARV adherence percentage by disclosure age (years).

A correlation was found between disclosure age and mean CD4 (β = -57.9, p < 0.0005; Fig 1), but not disclosure age and mean ART adherence (β = -0.023, p = 0.086; Fig 2). Multiple linear regression modeling (Table 3) was then used to look for differences in outcome variables related to the patient’s age of disclosure. A younger disclosure age (in years) predicted a higher mean CD4 count over the course of the patient’s clinical care (β = -68.11, p = 0.001, R^2^ = 0.2756) while controlling for sex, entry point to care, main caregiver, caregiver change, and total number of visits. No significant difference was found in mean ART adherence percentage with earlier ages (in years) of disclosure (β = -0.0322, p = 0.055) while controlling for the same variables. None of the control variables were found to be significantly correlated to either of the outcome variables.

**Table 3:** Associations between disclosure age (in years) and clinical outcome variables. Regression model controls for sex, entry point to care, total number of visits, main caregiver, and caregiver change during study. No control variables were shown to have a significant association with outcome variables. CI, confidence interval.

	β	*P*	R^2^	95% CI
***Mean ART adherence percentage***	-0.03223	0.055	0.2272	-0.06522–0.00077
	β	*P*	R^2^	95% CI
***Mean CD4***	-68.11074	**0.001**	0.2756	-106.444–-29.7775

## Discussion

The aim of this research was to determine whether disclosure of an HIV diagnosis to an adolescent has an effect on medical outcomes for ALHIV. This retrospective longitudinal study found an improvement in mean ART adherence with the act of disclosure to adolescents in Kericho, Kenya. Furthermore, we found an improved mean CD4 count with a younger age of disclosure (conversely, delayed disclosure predicts a lower mean CD4 count, as the correlation and regression models illustrate). Our findings are consistent with previous qualitative and cross-sectional studies that have described improved adherence with disclosure[8,11,12,14,18,22,26,29,30]. Far fewer longitudinal studies, such as ours, have looked at the associations between disclosure and adherence or outcomes over time[9,23]. Our study adds that a younger age of disclosure may have a beneficial effect on the clinical HIV outcome measure of CD4 count. It is among the first of its kind to look at adolescents’ outcomes over time, highlighting the importance of disclosure of an HIV diagnosis to ALHIV.

Translated into clinical terms, and based on our regression model, each year of disclosure delay is associated with a substantially lower mean CD4 count (68.11 cells/μL, 95% CI 29.78–106.4 cells/μL). This relationship between older age and lower mean CD4 count may be related to one of the reported rationales for disclosure of HIV infection to an adolescent—waiting for HIV disease progression and related illness before disclosing—that has been described previously[30]. This could indicate that we are missing an opportunity to *prevent* clinical decline through proactively involving an adolescent in his or her clinical care at an earlier age. Our finding of improved mean ART adherence percentage with disclosure to adolescent patients is consistent with the improved mean CD4 count seen with earlier disclosure.

The KDH disclosure process involves linking adolescents with HIV support groups, where peers with similar challenges and concerns may seek assistance. Although disclosure is generally a logical requisite to participate in ALHIV support groups, it is impossible to state which aspect or aspects of disclosure (e.g. the act of disclosure itself, the changes in communication among patients and caretakers, the shared experiences from peers at support groups) are responsible for improved adherence. There was no data in the medical record that recorded or described support group involvement or the extent of that involvement. ART adherence and health effects of adolescent peer group participation are interesting areas for future and continued research.

Our study has some limitations. Disclosure is difficult to define, and is a process more so than an absolute, yet our measurement was binary out of necessity. Although disclosure was consistently and systematically assessed at each clinic visit over the ten-year study period, there may have been inter-rater variability over time. It is also quite possible, particularly in the older adolescents, that patients were aware, or had a suspicion, of their HIV diagnosis even before they were formally told by their clinical teams and caretakers. It is difficult to know the length of such a transition period, or to know the effects of delayed disclosure on a suspecting adolescent.

As with many similar studies in low and middle income countries, lack of resources leads to gaps in data. This study was conducted before the World Health Organization’s 90-90-90 targets and push for viral load tracking as the desired clinical outcome, and we were unable to assess for an association between disclosure and viral load given the paucity of viral load assays completed during the study period. As sites similar to Kericho are able to consistently track viral loads in addition to adherence and CD4, future prospective studies are needed to look for links between disclosure and clinical outcomes in ALHIV.

In conclusion, our results support disclosure of an HIV diagnosis to an ALHIV in order to improve ART adherence and CD4 counts. Our findings may further indicate that disclosure should occur proactively, at younger ages and in developmentally appropriate ways, before CD4 decline; this is an area of interest for future research. This study adds to the body of growing literature that supports the importance of earlier disclosure to adolescents, and helps to inform the continued discussion on disclosure of an HIV diagnosis to adolescents.

## Supporting information

S1 File RV364 data for PLOS ONE. (CSV)
